# A Review about the Effect of Life style Modification on Diabetes and Quality of Life

**DOI:** 10.5539/gjhs.v4n6p185

**Published:** 2012-10-10

**Authors:** Prabha Shrestha, Laxmi Ghimire

**Affiliations:** 1 Faculty of Health, School of Nursing and Midwifery, Burwood Campus, Deakin University, Melbourne, Australia; 2 Department of Public Health, School of Medicine & Dentistry, University of Aberdeen, Aberdeen, Scotland, United Kingdom

**Keywords:** diabetes, quality of life, life style modification, diet, exercise

## Abstract

The aim of this review is to examine diabetes and quality of life improvements through modifying life style. The data was collected by reviewing published articles from PubMed, Medline, Web of Science, and Google open access publications. The review identified prevention strategies can reduce the risk and complications of diabetes. Life style modification in relation to obesity, eating habit, and physical exercise can play a major role in the prevention of diabetes. Nowadays, there has been progress in the development of behavioural strategies to modify these life style habits and it is not easy to accept for long term basis. If people maintain a balanced diet and physical exercise this can have real and potential benefits for their prevention and control of complications from chronic diseases particularly for cardiovascular risk and diabetes. Healthy life style may best be achieved through public private partnerships involving government, partners organizations, health services providers, community and people living with diabetes. Effective strategies to reduce the incidence of diabetes globally and assist in managing the disease are urgently required.

## 1. Introduction

Diabetes is a major public health problem globally with an increasing disease trend. A total of 366 million (8.3%) people lived with diabetes in 2011 and 4.6 million deaths were attributed to diabetes. The incidence is estimated to increase to double the 2011 data to 552 million in 2030 (International Diabetes Federation n.d.), if no action is taken. The diabetes epidemic is worse in developing Asian countries. Asian people are at significant risk of diabetes in comparison to western societies, because of their changing life style and consumption of white rice (Hu et al., 2012).

A major public health problem occurring is the increasing incidence of diabetes in Asian countries including Nepal and India. A study in 2009 reported a diabetes prevalence rate of 25.9% of the population in Kathmandu the capital city. The rate for men was 27.1% and women 24.8%, where as in India, the prevalence of diabetes is reported as 4.3% overall 4.4% in men and 4.5% in women (Yadav et al., 2012). Why the Nepalese context has a higher incidence rate than India despite a similar socio-cultural situation is unknown. A further study has yielded the incidence is higher in urban areas (22.8%) compared to rural context (20%) and men (16.6%) and women (22.4%) (Yadav et al., 2012). Despite differing reports of the magnitude of the problem, the evidence suggests that the rate of diabetes in Nepal is considerably higher than global data, which needs to be dealt with effectively.

## 2. Methods

### 2.1 Search Engine

An extensive review of published articles related to diabetes; prevalence, quality of life (QoL), prevention, diet and exercise were accessed from Web of Science, BMJ, BMC, Lancet, Diabetes care, etc. Additionally articles extracted from online sites, magazines, peer review articles, newspaper and open access materials in Google Scholar were accessed. The literature review covered more than thirty published materials from 1999 to 2012 focusing on diabetes prevention programs.

### 2.2 Data Abstraction and Analysis

The prevalence of diabetes, QoL, wellbeing, prevention and life style modification were the key words used for searching the web. We attempted to review diabetes prevention programme broadly rather than country specific. This paper focuses on prevention, delaying onset and control of diabetes complications by daily life style changes.

## 3. Results

Diabetes is a major contributing factor for overall health status, morbidity, mortality and QoL. Uncontrolled diabetes increases the number of serious health problems such as heart attack, stroke, blindness, kidney and peripheral blood vessel disease. Diabetes leads to a high risk of kidney disease (Mittal et al., 2010), pneumonia (Lepper et al., 2012), heart disease, high blood pressure and a higher death rate occurs in diabetes patients than non-diabetic patients. Another interesting outcome is that approximately 50% of tuberculosis patients are reported as having diabetes or being pre-diabetic (Viswanathan et al., 2012). All these health conditions result in reduced QoL.

### 3.1 Quality of Life

Quality of life varies with individuals, societies, people with diabetes and non-diabetes and it depends on diet and physical activity, controlling the disease complications and health improvements accomplished (Hu et al., 2010; Rubin & Peyrot, 1999; Venkataraman et al., 2012). Quality of life is known to affect mental, physical, social wellbeing and daily lives. The prevalence of depression is higher (24%) in diabetic than non-diabetic persons (17%) with significant differences in quality of life indices between depression with diabetes and non-diabetes (Goldney et al., 2004). Psychological distresses (depression, anxiety and sleep disturbances) can have a negative impact on QoL. The risk of depression is higher in diabetes, undiagnosed diabetes and impaired glucose metabolism that have serious threat to QoL (Nouwen et al., 2011). In addition, peripheral neuropathy complications affects health related QoL in diabetic as well as retinopathy, coronary disease, and kidney disease (Venkataraman et al., 2012). Thus it should be higher priority in prevention and control of diabetes.

Evidence has revealed that effective preventative program can help in reducing or delaying the incidence of diabetes and pre-diabetes and improving QoL. Age, sex, education, occupation, income, smoking, alcohol use, history of cardiovascular disease, physical activity, a large body mass index and waist circumference are associated with impaired fasting glucose and diabetes (Qin et al., 2010). It is known that diabetes can be prevented by modification to a healthy among high risk groups (Toumiletho et al., 2001; Penn et al., 2009).

### 3.2 Life Style Modifications

The life style of individual focusing on their diet, weight, physical activity, tobacco smoking and alcohol drinking, has identified that out of those factors weight loss is the major predictor in the prevention of diabetes. There are many effective approaches in the reduction of weight loss like low fat and calorie diets, high fibre and protein rich diets combined with regular exercise. A study shows that individualized dietary counselling, circuit type resistance training session and advice on increasing physical activity reduce the weight and risks of diabetes (Lindstrom et al., 2003). An individual with diabetes, achieving moderate weight loss with physical activity may control their blood sugar and improve insulin sensitivity (Klein et al., 2004). A small group weight loss session is important in prevention of diabetes (Almeida et al., 2011). A randomized control trial found significant weight loss in both hypo-caloric almond-enriched diet and hypo-caloric nut-free diet groups but comparatively smaller scale of weight loss in hypo-caloric almond-enriched diet (Foster et al., 2012).

A study reported that life style modification reduces diabetes incidence up to 55% (Penn et al., 2009), and delays disease progression (Heneghan et al., 2006) as well as managing diabetes symptoms successfully (Wei et al., 1999). Life style intervention (diet and exercise) can reduce the incidence of diabetes by 28-59% in impaired fasting glucose and or impaired glucose tolerance (Walker et al., 2010). A community based life style prevention measure reduced the fasting blood sugar level of diabetes and pre-diabetes by 25% and 11% respectively (Balagopal et al., 2008). The life style interventions were identified as more effective than anti-diabetic medicines. It has been reported that effective life style programs prevent diabetes by 58% where as Metformin only by 31% (Diabetes Prevention Program Research Group, 2002). Therefore life style change activity can be more effective, cheaper and safer than anti-diabetic medications (Gillies et al., 2007). Life style intervention is feasible in primary health care settings with a higher level of education significant in reduction of weight, waist measurements, glucose levels, lipid analysis and psychological distress (Laatikainen et al., 2007).

However, it is not always easy to replicate life style intervention programs in developing countries even in well funded health care systems, as it requires a co-ordinated effort among government, society and funding sources (Heneghan et al., 2006). Behaviour change to modify life style is another challenge in the prevention of diabetes as it requires considerable effort, motivation and time. Counselling can be a useful motive for people in changing their behaviour internally. A randomized controlled trial found that short term goal setting and problem solving techniques with social support and regular follow-up programs are useful to sustain life style behaviour modification (Pettman et al., 2008). The same study confirmed that delivery of group training by peers is more effective than the availability of guidelines to manage obesity and cardio-metabolic risk factors. Modification of dietary behaviour, physical activity and smoking behaviour can be sustained by combination of two counselling methods: motivational interviewing and problem solving treatment (Lakerveld et al., 2008).

#### 3.2.1 Dietary Therapy

Many policy makers are contemplating introducing a tax on unhealthy foods. For instance, Denmark introduces the ‘fat tax’ for high saturated fat (more than 2.3%) foods: butter, milk, cheese, pizza, meat, oil and processed foods (Mytton et al., 2012). Some scientists suggest that salt, sugar and refined carbohydrate should be treated the same as fat, as these are more harmful to health (BBC News, 2011). Hungary and France have introduced a junk food tax with sweetened drinks whilst Peru has planned to implement a similar tax. Additionally, there is no association between reducing sugar consumption and reducing the prevalence of obesity (Barclay & Brand-Miller, 2011).

#### 3.2.2 Physical Activity

Regular physical activity increases the functions of the body and reduces the risk of diabetes (Harvard School of Public Health, 2012). Well-structured physical activity is effective in reducing the incidence of diabetes and restoring normal glucose measures amongst high risk groups (Malkawi, 2012). An inactive physical life style may increase diabetes and impaired fasting glucose with low treadmill exercise related to elevation of diabetes impaired fasting glucose (Wei et al., 1999). Lack of physical activity excluding walking is a risk of diabetes (Qin et al., 2010).

A challenge exists in developing, implementing and evaluating effective low cost prevention programs at the local level. It has been reported that a single program for people with newly diagnosed diabetes does not make a difference in biomedical changes and life style outcomes over a three year period (Khunti et al., 2012). Although it showed signs of improvement in some health beliefs, how then to develop an effective diabetes education program that is culturally relevant in a developing country context is required.

## 4. Discussion

The review identified the effectiveness of life style interventions including diet plus exercise in preventing the incidence of diabetes or delaying the onset. Several studies identified; life style prevention strategies as more effective than medical treatment including Metformin to prevent and reduce diabetes symptoms (Diabetes Prevention Program Research Group, 2002). Diet plus exercise is the most effective preventative strategy in reducing the incidence of diabetes (Li et al., 2008; Walker et al., 2010). Another study found improvements in blood glucose control, body weight and insulin resistance in the intervention group with diet and diet plus activity with less use of anti-diabetic medicines. This was compared to a control group who received usual care at 6 months review (Greaves et al., 2011; Malkawi, 2012). Intervention is more effective with social support, follow-up, using self–setting behaviour change and self-monitoring (Greaves et al., 2011). Diet and exercise are the most important components of life; reducing weight, eating the right food and regular exercise are central to the management of diabetes rather than medical treatment (Echouffo-Techegul & Dagogo, 2012; Gillies et al., 2007). Choosing healthy food is important as is consuming appropriate food on a regular basis. These foods may reduce weight and blood glucose but most importantly they assist in reducing the risk of heart disease and high cholesterol. The use of a group setting and supportive ’peer’ leaders were found to be supportive with frequent clinical assessment suggested for future programs (Pettman et al., 2008).

## 5. Conclusion

This paper had identified that the increasing diabetes trend can be prevented. What is required is life style alteration that can prevent and reduce the risk of developing diabetes and its complications. It is essential that information about maintaining and controlling weight, dietary modification and regular exercise is provided through health education programs as well as promoting healthy behaviour in local health organizations, such as general practitioners, clinics and hospital waiting areas. It is also important to undertake more research on the preventative measures in the local context which promote healthy life styles therefore leading to improved quality of life for individuals and societies.
